# Place-Based Understanding of Social Isolation and Loneliness in U.S. Subsidized Senior Housing: A Scoping Review

**DOI:** 10.1093/geroni/igaf122.2786

**Published:** 2025-12-31

**Authors:** Jihye Baek, Byeongju Ryu, Sojung Park

**Affiliations:** Washington University in St. Louis, St. Louis, Missouri, United States; Washington University in St. Louis, Clayton, Missouri, United States; Washington University in St. Louis, St. Louis, Missouri, United States

## Abstract

Subsidized senior housing (SSH) provides affordable housing and social services to low-income older adults, promoting opportunities for social engagement. Despite these communal benefits, residents often experience social isolation and loneliness due to various individual and environmental challenges, including health issues, financial constraints, limited transportation options, and changes in social relationships. This scoping review examined factors associated with social isolation and loneliness through a socio-ecological framework. A systematic search of six electronic databases identified 12 studies, and a thematic analysis synthesized the evidence across multiple levels. At the individual level, residents facing physical and mental health challenges, as well as limited social skills and financial resources, faced a higher risk of social isolation and loneliness. At the interpersonal level, weakened family ties intensified social isolation, while peer relationships within SSH fostered belonging and mutual support. At the community and organizational levels, improved access to transportation, social spaces, and community cohesion served as protective factors against social isolation and loneliness. Societal factors, including the COVID-19 pandemic and changes in the digital environment, influenced social isolation and loneliness. Findings emphasize the interplay between personal and environmental factors, highlighting the need for multi-level interventions. Supporting individuals with limited personal resources while improving transportation access and community engagement may mitigate these challenges. Additionally, addressing broader societal factors, such as the impact of the pandemic and technological changes through context-specific strategies, can enhance residents’ social well-being. We emphasize that a contextualized understanding of social isolation and loneliness is crucial for developing effective interventions.

